# Periodontal Ligament Stem Cells: Current Status, Concerns, and Future Prospects

**DOI:** 10.1155/2015/972313

**Published:** 2015-03-16

**Authors:** Wenjun Zhu, Min Liang

**Affiliations:** ^1^Department of Periodontology, Guanghua School of Stomatology, Hospital of Stomatology, Sun Yat-sen University, 56 Ling Yuan Road West, Guangzhou, Guangdong 510055, China; ^2^Guangdong Provincial Key Laboratory of Stomatology, China

## Abstract

Periodontal ligament stem cells (PDLSCs), which reside in the perivascular space of the periodontium, possess characteristics of mesenchymal stem cells and are a promising tool for periodontal regeneration. Recently, great progress has been made in PDLSC transplantation. Investigators are attempting to maximize the proliferation and differentiation potential of PDLSCs by modifying culture conditions and applying growth factors. Nevertheless, problems remain. First, incomparability among different studies must be minimized by establishing standard guidelines for culture and identification of PDLSCs. Notably, attention should be paid to the biological safety of PDLSC transplantation. The present review updates the latest findings regarding PDLSCs and discusses standard criteria for culture and identification of PDLSCs. Finally, the review calls for careful consideration of PDLSC transplantation safety.

## 1. Introduction

Periodontitis is an infectious and inflammatory oral disease that leads to periodontal tissue destruction and is the main reason for tooth loss [1]. Dentists have succeeded in controlling periodontal inflammation through conventional therapies but have failed to restore the damaged periodontium. The aim of periodontal regenerative treatment is to restore the physiological function of teeth by rebuilding periodontal supporting tissues including alveolar bone, gingiva, periodontal ligaments (PDL), and the cementum. With tissue regeneration, damaged periodontal tissues can be repaired via application of stem cells, growth factors, or an extracellular matrix scaffold [2]. To date, several types of stem cells have been investigated for periodontal regeneration, which comprise mesenchymal stem cells (MSCs), embryonic stem cells (ESCs), and induced pluripotent stem cells (iPSCs). MSCs are gaining acceptance for use in periodontal regeneration because their application is not subject to ethical issues as are ESCs [2]. MSCs were initially discovered in bone marrow, and bone marrow mesenchymal stem cells (BMMSCs) were found to promote periodontal regeneration when transplanted into periodontal osseous defects [3]. Considering the inconvenience of obtaining BMMSCs, such as pain and trauma from invasive bone marrow aspiration and low numbers of harvested cells [4], subsequent attempts to harvest MSCs have been from dental-derived tissues, such as the periodontal ligament [5], gingiva [6], dental follicles [7], dental pulp [8], apical papilla [9], and human exfoliated deciduous teeth [2, 10]. In 2004 Seo et al. [5] successfully isolated multipotent periodontal ligament stem cells (PDLSCs) from human impacted third molars and these cells could differentiate into periodontal ligaments, alveolar bone, cementum, peripheral nerves, and blood vessels [11–13]. Currently improving the regenerative ability of PDLSCs is of interest to investigators, and the present review reveals the latest findings regarding PDLSCs and elaborates on the difficulties and concerns about PDLSCs in terms of cell culture, identification, and biological safety.

## 2. Basic Properties of Periodontal Ligament Stem Cells

Periodontal tissues are reported to arise from migrated neural crest cells during tooth development [14]. However, PDLSCs obtained from mature periodontal ligaments possess stem cell properties similar to MSCs rather than neural crest cells [15]. More specifically, PDLSCs express MSC surface markers (CD105 [16–20], CD90 [16–18, 20, 21], and CD73 [19, 22]) but lack expression of CD45, CD34, and CD14 or CD11b, CD79a, or CD19 and HLA class II [20]. Moreover, PDLSCs located in the perivascular wall of periodontal ligaments share similarities with pericytes in morphology, differentiation potential, cell phenotype (expression of pericyte-associated markers CD146, neural/glial antigen-2 and CD140B), and the ability to form capillary-like structures* in vitro* [23]. Recent studies show that MSCs obtained from various tissue types (brain, lung, liver, kidney, muscle, skin, and bone marrow) also exhibit pericyte characteristics [24, 25].

In addition, PDLSCs have an immunomodulatory ability that is comparable to bone marrow MSCs [18]. First, PDLSCs possessed low immunogenicity due to the absence of HLA-II DR or T cell costimulatory molecules (CD80 and CD86) [26]. Secondly, PDLSCs inhibited proliferation of allogeneic T cells through upregulation of cyclooxygenase-2 (COX-2) and prostaglandin E2 (PGE2) [26]. Surprisingly, after osteogenic induction, the inhibitory effect of PDLSCs on T cell proliferation was intact [27]. Third, PDLSCs suppressed B cells proliferation, differentiation, and migration through cell-to-cell contact, which was mediated by programmed cell death protein-1 [28]. The low immunogenicity and immunosuppressive effects on T and B cells allow use of allogeneic PDLSCs in periodontal regeneration. Indeed, allogeneic PDLSCs have been tested in a sheep [29] and a swine [26] model, and the therapeutic effects of allogeneic PDLSCs were equal to those of autologous PDLSCs.

In summary, PDLSCs are a subpopulation of MSCs located in the perivascular space which share similarities with pericytes. However, there is no standard criterion for the identification of PDLSCs, which leads to incomparability among study data. Although using MSC criteria [30] for identifying PDLSCs may reduce these differences, they do not emphasize specific properties of PDLSCs. Thus, a standard criterion is needed for PDLSC identification.

## 3. Factors That Influence Stem Cell Properties of PDLSCs

Various factors have been shown to regulate stem cell properties of PDLSCs, including tissue origin, age of donor, inflammatory condition, culture method, and growth factors.

### 3.1. Tissue Origin

PDLSCs were collected mainly from the midthird portion of the root surface after permanent tooth extraction. However, Wang and colleagues [17] demonstrated that some PDL tissue remained in the alveolar socket. PDLSCs isolated from the alveolar socket—alveolar bone derived PDLSCs (a-PDLSCs)—were compared with conventional root surface-derived PDLSCs (r-PDLSCs) and had higher proliferative ability, as well as stronger osteogenic and adipogenic differentiation potential than r-PDLSCs.

Deciduous teeth have been gaining attention for PDLSC sources [21, 31, 32]. PDLSCs derived from deciduous teeth (d-PDLSCs) had greater proliferation, stronger adipogenic potential, and osteogenic potential than PDLSCs derived from permanent teeth (p-PDLSCs) [31, 32]. D-PDLSCs could also form a cementum-PDL structure when implanted in a nude mouse [32]. These observations suggested that d-PDLSCs may be more suitable than p-PDLSCs for periodontal regeneration, but Song and colleagues' findings did not agree with this [21]. No significant differences were documented in their studies between d-PDLSCs and p-PDLSCs in terms of proliferation rate, expressions of stem cell markers, or* in vitro* differentiation potential. P-PDLSC transplants formed a more typical cementum/PDL-like tissue and expressed more cementum/PDL-related genes (CP23 and collagen XII) than did d-PDLSCs transplants. Thus, the PDLSC type that is best for periodontal regeneration is uncertain as is d-PDLSCs safety.

Questions remain about whether d-PDLSCs can cause root absorption when they contact the root surface of permanent teeth. Jin's group [33] discovered that PDLSCs derived from resorbed primary teeth expressed increased RUNX2, which upregulated RANKL and downregulated OPG at both the mRNA and protein levels. These imbalances between RANKL and OPG finally led to osteoclast differentiation and root absorption. Thus, d-PDLSCs from resorbed primary teeth may cause unexpected activation of osteoclasts when used in periodontal regeneration but this requires confirmation.

Recently additional sources of PDLSCs have been identified. Lee's group [21] revealed that PDLSCs obtained from periodontal ligaments of supernumerary teeth had better colony-forming efficiency than BMMSCs and could differentiate into adipocytes and osteoblasts. Furthermore, PDLSCs isolated from periodontal granulation tissue [34, 35] in periodontitis patients expressed Stro-1 and CD146 and improved new bone formation when transplanted in mouse calvarias defects. Even so, the potential risks of infected tissue-derived PDLSCs are a concern because the effects of pathogenic microorganisms on PDLSCs are largely unknown. For instance, LPS from* Porphyromonas gingivalis* (the main pathogen of chronic periodontitis) severely inhibited osteogenic differentiation and promoted expression of proinflammatory cytokines (IL-1*β*, IL-6, and IL-8) in human PDLSCs [36], and the duration of such inhibitory effects remains to be investigated.

Inflammation in periodontal tissue not only diminishes bacteria, but also destroys periodontal supporting tissues. Growing evidence suggests that inflammation also hampers the regenerative ability of PDLSCs. PDLSCs derived from inflamed periodontal tissues (i-PDLSCs) had greater proliferation [37] and faster migration [38] but had lower osteogenic capacity [37] and lower cementogenesis potential compared with PDLSCs obtained from healthy periodontal tissue (h-PDLSCs). These inhibitory effects were due to activation of NF-*κ*B [39], upregulation of *β*-catenin, and activation of the canonical Wnt pathway [16, 37]. Also, immunomodulatory effects of i-PDLSCs were suppressed [40]. The inhibitory effect of i-PDLSCs on T cell proliferation was significantly diminished, and the inductions of CD4 + CD25 + FOXP3 + regulatory T cells and IL-10 secretion were also hampered when i-PDLSCs were cocultured with stimulated peripheral blood mononuclear cells.

At present, tooth extraction is inevitable during PDLSC isolation and this is an obstacle for using autologous PDLSC transplantation for patients who do not need tooth extraction. In comparison, MSCs from gingiva (GMSCs) are easily accessible in the oral cavity [6] and have been documented to regenerate cementum, alveolar bone, and periodontal ligament in a dog model [41]. GMSCs isolated from normal gingiva and cyclosporine-A induced hyperplastic gingiva [42] were similar with respect to self-renewal and multipotency. Moreover, both cell types induced CD4 + CD25 + FOXP3 + regulatory T cells and inhibited proliferation of peripheral blood mononuclear cells, similar to PDLSCs. In addition, Chen's team investigated the influence of inflammatory cytokines TNF-*α* and IL-1*β* on GMSCs and PDLSCs and measured osteogenic differentiation potential [43]. They found that osteogenic differentiation of GMSCs and PDLSCs was partially inhibited by the inflammatory environment. However, GMSCs were more resistant to TNF-*α* and IL-1*β* compared with PDLSCs, even though the osteogenic area formed by PDLSCs was greater than that of GMSCs under inflamed or normal conditions.

In summary, PDLSCs can be obtained not only from healthy permanent teeth and deciduous teeth, but also from supernumerary teeth and inflamed granulation tissue. Of note, these expendable periodontal tissues may become important future sources of stem cells. To maximize the therapeutic effects of PDLSCs, studies are needed to differentiate biological properties of PDLSCs obtained from different tissues and methods are required for reducing inflammation.

### 3.2. Donor Age

Donor age also affects stem cells. PDLSCs obtained from aged donors had less regenerative capacity compared with those from young donors [44]. Therefore, the effects of aging on stem cell properties, especially prior to autologous application of PDLSCs in elderly patients, warrant attention. Zhang and coworkers [45] compared biological features of PDLSCs obtained from donors at different ages and found that proliferation and migration ability and differentiation potential of PDLSCs decreased as donor age increased. Moreover, PDLSCs in aged groups (older than 41 years) expressed less Stro-1 and CD146 than young donors and failed to form cementum-PDL-like structures* in vivo*, indicating that the number and regenerative ability of stem cells decreased with increasing donor age.

Thus, donor age is relevant to autologous PDLSC transplantation because chronic periodontitis patients are usually older than 40 years of age when the regenerative capacity of PDLSCs may have been compromised. Zheng's laboratory [44] reported that PDLSC proliferation and differentiation from aged donors were partially restored after exposure to young donor PDLSC-conditioned medium. In contrast, aged donor PDLSC-conditioned medium suppressed the regenerative capacity of young donor's PDLSCs and inhibited cementum-PDL-like structure formation* in vivo*. Soluble factors, especially growth and differentiation factors, in the conditioned medium affected stem cell properties as well; therefore, studies are needed to investigate the effects of various soluble factors secreted by PDLSCs. Finally, techniques are needed for the restoration and improvement of PDLSC regenerative capacity in elderly patients.

The replicative senescence of MSCs is inevitable, especially for cell culture. The loss of proliferative and regenerative capacity of MSCs is due to loss of telomere length during cell division. According to Baxter et al. [46], MSCs* in vivo* have already undergone substantial telomere erosion at the speed of 17 base pairs per year. The accumulation of telomere shortening may finally cause growth arrest and loss of multipotency [47], which is irreversible due to the absence of human telomerase reverse transcriptase (hTERT) in MSCs [48, 49]. Based on this theory, Fujii and colleagues [50] established three immortalized human PDL fibroblast cell lines with simian virus40 T-antigen and hTERT transfection. These immortalized PDL cells maintained high proliferative activity after 120 population doublings, whereas normal PDL cells stopped proliferating after 10 to 20 population doublings. The immortalized cell lines were similar to normal PDL cells in terms of gene expression and multipotency, suggesting their utility for studying biological mechanisms of human periodontal ligament cells.

Considering that postnatal stem cells will gradually lose their stem cell properties, pluripotent embryonic stem cells with an almost infinite life span have been investigated for regeneration of periodontal defects in a porcine model [51]. Porcine GFP-expressing ESCs carried by a collagen matrix were delivered to periodontal furcation defects and they formed cementum and periodontal ligament three months after transplantation. Because ESC research is often hampered by ethical concerns, somatic cells can be transformed into iPSCs, which are equivalent to ESCs in many aspects, by transfecting OCT3/4, SOX2, KLF4, and C-MYC [52] or* OCT4*,* SOX2*,* NANOG*, and* LIN28* [53]. Duan and coworkers [54] reported that the application of iPSCs with enamel matrix derivatives and a silk scaffold improved periodontal regeneration in a nude mouse model. Of note, iPSCs maintain an epigenetic memory of their tissue of origin, which modifies the differentiation potential of specific iPSCs [55]. For example, iPSCs derived from nonhematopoietic cells such as neural progenitors and fibroblasts had reduced blood-forming potential because of residual methylation at loci required for hematopoietic fate [55]. Hence, Wada and colleagues [56] generated iPSCs from human gingival and periodontal ligament fibroblasts. At this time, it is uncertain whether iPSCs from gingiva or PDL are better than traditional GMSCs or PDLSCs and more investigations are necessary to evaluate the efficacy and safety of these stem cells.

### 3.3. Culture Methods and Conditions

Improvements have been made in cell culture methods and conditions to expand PDLSCs rapidly without losing their stemness. For primary culture of PDLSCs, both outgrowth and enzymatic dissociation methods were feasible [57]. However, PDLSCs cultured by enzyme digest methods had greater proliferation rates, better colony-forming efficiency, and stronger differentiation capacity than outgrowth PDLSCs. Furthermore, the successful rate of primary culture was greater with type I collagenase and dispase (*n* = 30, 96.7%) together than with using trypsin and EDTA (*n* = 11, 72.7%) [58]. Hence, type I collagenase and dispase are recommended for primary culture of PDLSCs.

The culture medium, which is often ignored, also affects biological features of PDLSCs. Two media are extensively used to culture MSCs and PDLSCs: Dulbecco's minimum essential medium (DMEM) and *α*-minimum essential medium (*α*-MEM) containing L-glutamine and L-ascorbicacid-2-phosphate. Both *α*-MEM and DMEM can maintain stem cell phenotypes (expression of Stro-1, CD146, CD105, and CD44) of PDLSCs within passage 8. However, PDLSCs cultured in *α*-MEM had greater proliferation rates and stronger osteogenic potential than PDLSCs cultured in DMEM [59]. This may be due to more amino acids, vitamins, and nucleotides in *α*-MEM than in DMEM. Thus, *α*-MEM is more suitable for PDLSCs culture than DMEM.

Cells are usually cultured at 20% oxygen during expansion* in vitro*. Nevertheless, hypoxia seems to be the physiological microenvironment for stem cells [60]. Expression of pluripotency markers (Oct-4, Sox-2, and c-Myc) and the differentiation potential of PDLSCs were significantly increased after culture under 2% oxygen [61]. Additionally, the osteogenic potential of PDLSCs was promoted under hypoxia (2% O_2_) via activation of p38 and ERK 1/2 signaling pathways [62]. Thus, hypoxia facilitates the maintenance of multipotency in PDLSCs.

Primary cultures of PDLSCs yielded small cell numbers [58] (average 1,250 cells), which is less than needed to generate a cell sheet for periodontal regeneration (at least 4 × 10^6^ cells) [19]. Thus, PDLSCs must proliferate at least 12 population doublings before application. Therefore, expanding PDLSCs* in vitro* without losing their stemness is important. Prockop's group [63] suggested that MSCs plated at low density generated more cell doublings than those seeded at high density. Similarly, Iwata and colleagues [58] reported that PDLSCs seeded at a low density (50 cells/cm^2^) proliferated far more rapidly than those seeded at a relatively high density (500 and 5,000 cells/cm^2^). The colony-forming efficiency of PDLSCs seeded at a low density increased with passage (colony-forming efficiency *P*_5_ > *P*_3_ > *P*_1_), implying that seeding cells at a low density may exclusively select highly proliferative and replicative PDLSCs [63].

### 3.4. Growth Factors

Various growth factors have been tested for modification of stem cell properties of PDLSCs and in this review, we concentrate on the sequential use of growth factors.

Recently, efforts to keep PDLSCs undifferentiated at early stages of cell culture have been made to ensure better multipotency of stem cells to differentiate into osteoblasts/cementoblasts and fibroblasts at later culture stages. According to Liu et al.'s research [64], nuclear expressions of Sox-2 and Oct-4 in PDLSCs were maintained until passage 3. Meanwhile, Sox-2 and Oct-4 mRNA in PDLSCs decreased and disappeared after passage 3. Surprisingly, bone morphogenetic protein-4 (BMP-4) not only could enhance proliferation, but also could reverse the decrease in Sox-2 and Oct-4 expression and promote their nuclear translocation even at passage 7. The beneficial effects of BMP-4 on PDLSCs may be due to the significant overlap in responsive genes between BMP-4 and Oct-4 [65]. BMP-4 may be an effective way to maintain the stemness of PDLSCs during a long-term culture.

As the periodontium is comprised of cementum, alveolar bone, and the functional periodontal ligaments between them, so use of various growth factors to induce PDLSCs differentiation into different directions is of interest. For example, BMP-2 and -7 and vascular endothelial growth factor (VEGF) have been verified to enhance osteogenic differentiation of PDLSCs and promote the repair of bony defect in animal models [66–69]. In contrast, transforming growth factor-*β*1 (TGF-*β*1) and its downstream protein connective tissue growth factor (CTGF) accelerated fibroblastic differentiation of PDLSCs through upregulation of type I collagen, *α*-smooth muscle actin, and periostin [70–72]. Furthermore, fibroblast growth factor 2 (FGF-2) promoted proliferation of PDLSCs but reversed the beneficial effects of BMP-2 and VEGF on osteogenic differentiation [68]. Unexpectedly, sequential use of FGF-2 followed by BMP-2 resulted in more bone formation than use of BMP-2 or FGF-2 alone [69]. Similarly, sequential use of FGF-2 followed by TGF-*β*1 also promoted fibroblastic differentiation of PDLSCs.

These data suggest that stem cells at different stages may need different types of growth factors to support their proliferation or differentiation. Sequential use of growth factors appears promising and effective for improving stem cell regeneration, but interactions among various growth factors have not been studied thoroughly.

## 4. Concerns and Advice regarding PDLSC Applications

**Figure 1:**
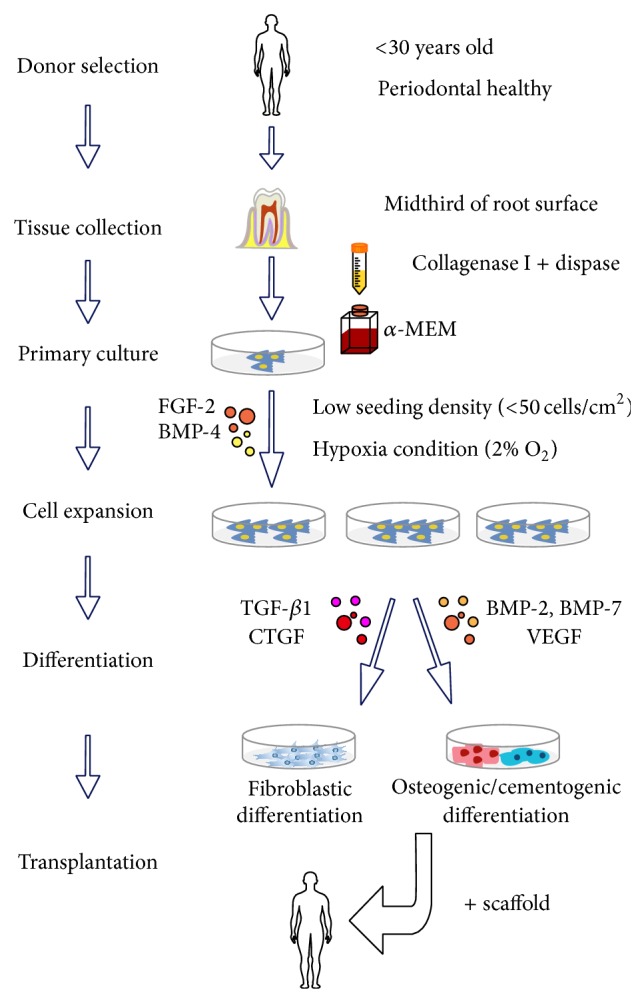
Optimized conditions for isolation and culture of periodontal ligament stem cells. This represents an improved protocol for isolation and culture of PDLSCs. Optimal culture methods and conditions such as using enzymatic digestion, *α*-MEM, and seeding cells at low density under hypoxia are recommended. Sequential use of different growth factors is effective for cell expansion and differentiation.

**Figure 2:**
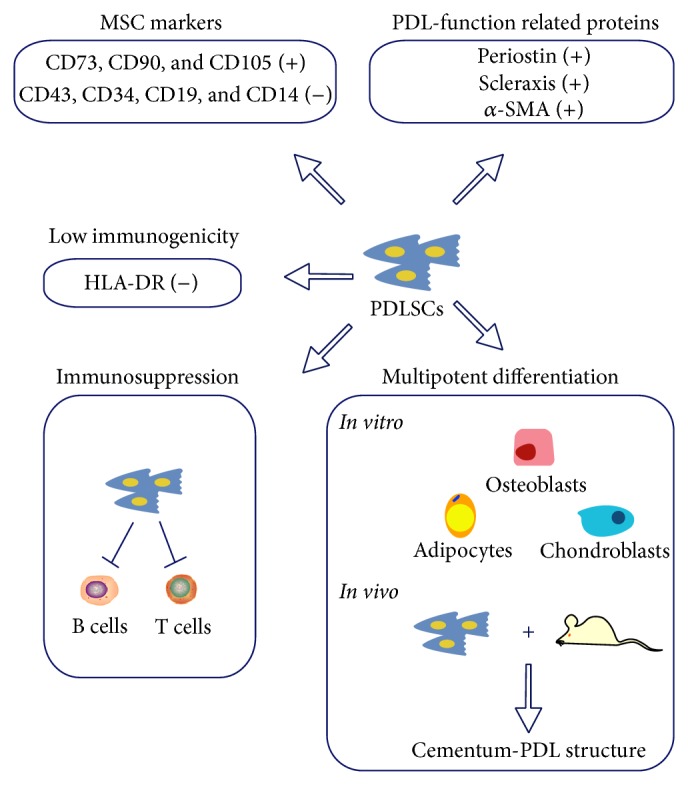
Criteria for identification of periodontal ligament stem cells. PDLSCs share similarities with MSCs in terms of antigenic phenotype and multipotent differentiation potential* in vitro*. In addition, PDLSCs also possess low immunogenicity and immunosuppressive ability, similar to MSCs. However, PDLSCs differ from MSC derived from other tissues or organs, such as expression of PDL-function related proteins (periostin, scleraxis, and *α*-SMA) and forming a cementum-PDL structure* in vivo*.

Recently, information about PDLSCs has expanded, but no standard protocol for PDLSC culture and identification is available and this leads to studies that cannot be compared. A consensus about isolating, culturing, identifying, and using PDLSCs is needed. Next, we have attempted to offer such a standard for isolation and identification of PDLSCs based on our review of the literature (Figures 1 and 2).

First, young patients (younger than 30 years of age) with a healthy periodontium are suitable candidates because the detrimental effects of age and inflammation on stemness and immunomodulation of PDLSCs can be avoided or at least alleviated. The decision between permanent or primary teeth for sourcing PDLSCs is unclear. Considering primary culture of PDLSCs, an enzyme digest method with type I collagenase and dispase is better than the outgrowth method. Moreover, hypoxia (2% O_2_), *α*-MEM, and a low seeding density (50 cells/cm^2^) enhance proliferation and maintain stemness of PDLSCs.

Various growth factors can improve the regenerative capacity of PDLSCs, and FGF-2 is good for modulating the effects of other growth factors. FGF-2 and its receptors (FGFR1–4), which are single-pass transmembrane proteins with tyrosine kinase activity [73], have been implicated in self-renewal and differentiation of embryonic stem cells [74] and MSCs [75]. In a dog and primate model [76, 77], a single topical application of FGF-2 in a gel carrier successfully regenerated the cementum, alveolar bone, and functional periodontal ligaments with Sharpey's fibers in class II furcation bone defects. Murakami's group [78] also discovered that FGF-2 can promote alveolar bone regeneration in periodontitis patients. However, continuous supplementation of FGF-2 severely inhibited ALP activity and calcified nodule formation of PDLCs [79], indicating that the time duration of FGF-2 is critical. In fact, topically applied FGF-2 only lasted one week according to tracer experiments with radiolabeled FGF-2 [79]. In addition, Maegawa and colleagues [69] tested different strategies for sequential use of FGF-2 and BMP-2 on BMMSCs, and they found that 6 days of supplementation of FGF-2 followed by 6 days of application of BMP-2 offered maximal mineralization. Either shortened or prolonged pretreatments with FGF-2 hampered the synergetic effects of FGF-2 and BMP-2 on osteogenesis.

Possible mechanisms for FGF-2 affecting osteogenesis may be several. FGF-2 increases the growth rate and the life span of BMMSCs and maintains their multipotency by inhibiting cellular differentiation, and this inhibitory effect is reversible after withdrawal of FGF-2 [79, 80]. Topically applied FGF-2 efficiently increases the number of Stro-1^+^/CD146^+^PDLSCs without affecting their stemness [81], and they can regenerate damaged tissues after FGF-2 disappears. Also, FGF-2 enhanced osteogenic effects of BMP-2 on PDLSCs and MSCs by upregulating expression of BMP receptor-1B [82] and stimulating VEGF secretion [71]. Other mechanisms that contribute to synergetic effects of these growth factors remain to be explored.

At this time, there is no specific marker for identifying PDLSC through labeling. Therefore, MSC characteristics, especially PDL functionally related properties of PDLSCs, should be stressed. First, PDLSCs must meet minimal criteria for defining MSCs. Briefly, PDLSCs express CD105, CD90, and CD73 (positive rate > 95%) but lack expression of CD45, CD34, and CD14 or CD11b, CD79a, or CD19 and HLA class II (positive rate < 2%). Other MSC markers such as Stro-1 and CD146 are useful indicators of immature stem cells, even though they are not PDLSC-specific. It is reported that PDLSCs positive for Stro-1 and/or CD146 have greater colony-forming efficiency and osteogenic potential than negative cells [83]. Also, several proteins related to the function of periodontal ligaments may be potential markers for PDLSCs identification. Periostin, which is also expressed by PDLSCs [58, 84–87], is an extracellular matrix protein [88] that helps with maintenance of periodontal ligament homeostasis [89]. Loss of periostin by gene knockout caused severe destruction of both periodontal ligaments and alveolar bone after exposure to mechanical stresses [90]. Recently Murakami's group [89] discovered an isoform of periostin that was predominantly expressed in periodontal ligaments rather than other tissues and organs (skin, lung, and heart). This PDL-specific isoform of periostin activated focal adhesion kinase (FAK) by binding to integrin *α*V*β*3 and eventually enhanced ALP activity and osteogenic differentiation of periodontal ligament cells. Hence, periostin is a PDL functionally related marker that can be used to identify PDLSCs. Scleraxis, a protein that is necessary for differentiation and maintenance of tendons and ligaments, is expressed by PDLSCs [17, 87, 91] as well. In scleraxis-null mice model [92], force-transmitting tendons and heart valves [93] were severely affected. Furthermore, upregulation of scleraxis was observed in rat bone marrow MSCs under mechanical stretching [94]. Similarly, scleraxis mRNA was increased when periodontal ligament cells were subjected to cyclic mechanical loading [95], indicating that scleraxis participates in tissue homeostasis under physical load. Moreover, *α*-smooth muscle actin (*α*SMA) proved to be a useful marker for identification of stem cells in the periodontium [96]. *α*SMA-positive cells residing in perivascular areas of PDL could differentiate into fibroblasts, osteoblasts, and cementoblasts during the healing process after PDL injury in a mouse model [97], which is consistent with the distribution and regenerative ability of PDLSCs. Thus, these three PDL functionally related markers (periostin, scleraxis, and *α*SMA) may be valuable for identifying PDLSCs.

In addition, the multipotent differentiation potential of PDLSCs should be verified not only* in vitro* (to differentiate into osteoblasts, adipocytes, and chondroblasts in differentiation medium) but also* in vivo* (to form a cementum-PDL-like structure in immunodeficient mice), and the latter is more convincing. According to Seo et al.'s research [5], five of thirteen strains of PDLSCs isolated by Stro-1 labeled magnetic beads could not generate mineralized or PDL-like tissues in immunocompromised beige mice, indicating that expression of the MSC marker Stro-1 does not guarantee periodontal regenerative capacity* in vivo*. Finally, the immune characteristics of PDLSCs are important. Immunological properties of MSCs can be altered under certain culture conditions. For example, FGF-2 enhanced the immunosuppressive potential of MSC* in vivo* [98], whereas inflammation suppressed immunosuppressive ability of PDLSCs [40]. Thus, immune properties of PDLSCs should be verified, especially for those to be used in allogeneic transplantation.

PDLSCs are usually obtained from young donors during tooth extraction for orthodontic reasons, but many times, the extracted teeth are discarded as medical waste because periodontal regeneration is rarely needed in young patients. Fortunately, periodontal ligaments or PDLSCs from these donors can be cryopreserved. After thawing, the PDLSCs retain their regenerative capacity [99, 100] and immunosuppressive ability and successfully regenerate periodontal tissues in a swine model [26]. Thus, cryopreservation may increase the convenience of allogeneic PDLSC transplantation.

**Figure 3:**
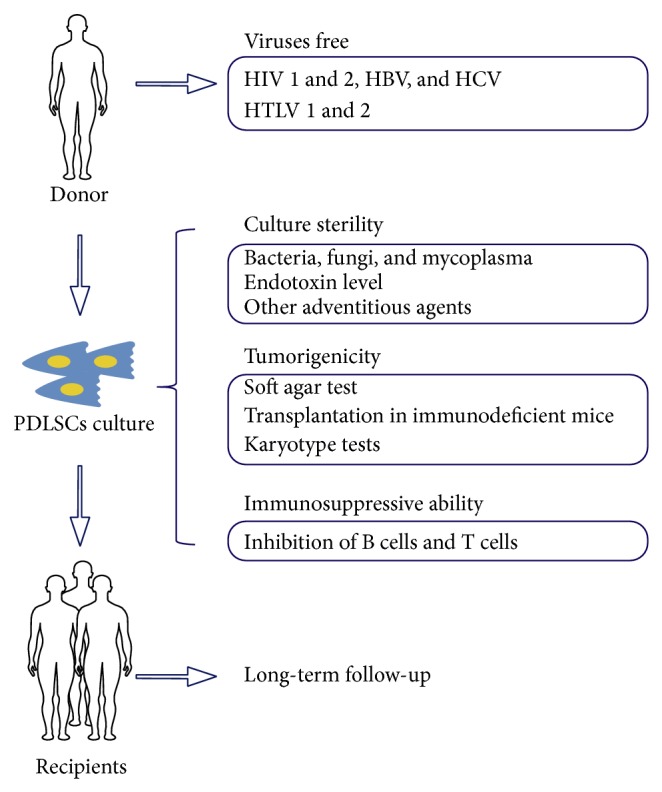
Biological safety during application of periodontal ligament stem cells. Donors must be free of viral infection to prevent cross infection between donor and recipient. Also, contamination by pathogenic microorganisms and adventitious agents during culture should be excluded. Next, the risk of tumor formation must be evaluated before PDLSC transplantation. Also, immunosuppressive ability should be tested to ensure the safety of allogeneic PDLSC transplantation. Finally, long-term follow-up of recipients is necessary to study the fate of PDLSCs and to minimize future risks after transplantation.

The biological safety of PDLSC transplantation, especially allogeneic transplantation, also requires consideration (Figure 3). First, PDLSCs must be free of contamination during isolation, culture, and delivery. Cell sheets and donor cells should be tested for viruses including human immunodeficiency virus (HIV) types 1 and 2, hepatitis B virus (HBV), hepatitis C virus (HCV), and human T-cell leukemia virus (HTLV) types 1 and 2 [101]. PDLSCs should be free of contamination by bacteria, fungi, and mycoplasma at the beginning and at the end of cell culture [102]. Further tests for adventitious agents [103] such as unexpected viruses or other substances of animal origin may also be necessary [101]. Efforts to reduce exogenous agents during cell culture have been attempted. For example, Tarle and coworkers [104] reported that PDLSCs cultured in a chemically defined, serum-free media (K-M) maintained their multipotency compared with PDLSCs cultured in *α*-MEM with 10% FBS. Recently, another xeno-free culture medium was investigated by Zini's [105] group, and this medium contained human albumin, recombinant human insulin, pasteurized human transferrin, HEPES, and L-glutamine, and it maintained genomic stability and multipotency of human PDLSCs. Also, autologous serum is suggested instead of fetal bovine serum during cryopreservation [106].

Stem cell transplantation also has tumorigenic potential. Amariglio and coworkers [107] reported that a patient who received transplantation of human fetal neural stem cells was diagnosed with a multifocal brain tumor that was derived from at least two donors. Although no case of MSC transplantation-related tumor formation has been reported, this risk must be considered before PDLSC transplantation. Yoshida and coworkers [108] suggested that both* in vitro* (soft agar test) and* in vivo* (transplantation in immunodeficient mice) tumorigenicity and karyotype tests (G-banding staining and multiplex fluorescence* in situ* hybridization) should be carried out before PDLSC transplantation.

Recently, immune rejection of autologous undifferentiated iPSCs transplantation in murine models was reported and data show that differentiation of iPSCs resulted in a loss of immunogenicity leading to immune tolerance. In contrast, undifferentiated iPSCs elicited a different immune response with high lymphocytic infiltration and elevated IFN-*γ* secretion [109], indicating that induction of* in vitro* differentiation may alter stem cell immune properties. Even though PDLSCs had intact immunosuppressive capacity prior to and after osteogenic induction [18, 27], the immune properties of PDLSCs should be tested once more prior to transplantation to ensure safety.

Finally, a continuous follow-up of recipients is imperative because long-term transplanted PDLSCs have not been studied. Gronthos's group [110] discovered that autologous PDLSCs labeled with BrdU were detectable 8 weeks after transplantation in an ovine periodontal defect. However, another experiment carried out by Kim and colleagues [111] had different results: donor cells from lacZ transgenic ROSA26 mice expressed blue color after X-gal staining, which was different from the host cells, and no donor cells were detected in the periodontal ligament space two weeks after allogeneic teeth transplantation. At present, the fate of PDLSCs after local transplantation are unclear, and the length of PDLSCs survival as well as their distribution should be confirmed by more reliable methods.
